# Supplementary material for: The Safety and Tolerability of a Potential Alginate-Based Iron Chelator; Results of A Healthy Participant Study
Source: Nutrients. 2019 Mar 21;11(3):674. doi: 10.3390/nu11030674 (PMC6471009; doi:10.3390/nu11030674)
Supplement: Supplementary file 1 [file nutrients-11-00674-s001.pdf]

Table 1. M-SHIME nutritional feed components.

Component	Amount (g/L)
Arabinogalactan	1.2
Pectin	2.0
Xylan	0.5
Glucose	0.4
Yeast Extract	3.0
Special Pepton	1.0
Mucin	2
L-cystein-HCl	0.5
Starch	4.0

1. **How often has the feeling of fatigue or being tired and worn out been a problem for you during the past 2 weeks?** Answer Value:
   - ☐ All of the time 1
   - ☐ Most of the time 2
   - ☐ A good bit of the time 3
   - ☐ Some of the time 4
   - ☐ A little of the time 5
   - ☐ Hardly any of the time 6
   - ☐ None of the time 7
2. **How often during the last 2 weeks have you delayed or canceled a social engagement because of your bowel problem?**
   - ☐ All of the time 1
   - ☐ Most of the time 2
   - ☐ A good bit of the time 3
   - ☐ Some of the time 4
   - ☐ A little of the time 5
   - ☐ Hardly any of the time 6
   - ☐ None of the time 7
3. **As a result of your bowel problems, how much difficulty did you experience doing leisure or sports activities during the past 2 weeks?**
   - ☐ A great deal of difficulty; activities made impossible 1
   - ☐ A lot of difficulty 2
   - ☐ A fair bit of difficulty 3
   - ☐ Some difficulty 4
   - ☐ A little difficulty 5
   - ☐ Hardly any difficulty 6
   - ☐ No difficulty; the bowel problem did not limit sports or leisure activities 7
4. **How often during the past 2 weeks have you been troubled by pain in the abdomen?**
   - ☐ All of the time 1
   - ☐ Most of the time 2
   - ☐ A good bit of the time 3
   - ☐ Some of the time 4
   - ☐ A little of the time 5
   - ☐ Hardly any of the time 6
   - ☐ None of the time 7
5. **How often during the past 2 weeks have you felt depressed or discouraged?**
   - ☐ All of the time 1
   - ☐ Most of the time 2
   - ☐ A good bit of the time 3
   - ☐ Some of the time 4
   - ☐ A little of the time 5
   - ☐ Hardly any of the time 6
   - ☐ None of the time 7
6. **Overall, in the past 2 weeks, how much of a problem have you had with passing large amounts of gas?**
   - ☐ A major problem 1
   - ☐ A big problem 2
   - ☐ A significant problem 3
   - ☐ Some problem 4
   - ☐ A little trouble 5
   - ☐ Hardly any trouble 6
   - ☐ No trouble 7
7. **Overall, in the past 2 weeks, how much of a problem have you had maintaining or getting to the weight you would like to be?**
   - ☐ A major problem 1
   - ☐ A big problem 2
   - ☐ A significant problem 3
   - ☐ Some problem 4
   - ☐ A little trouble 5
   - ☐ Hardly any trouble 6
   - ☐ No trouble 7
8. **How often during the past 2 weeks have you felt relaxed and free of tension?**
   - ☐ All of the time 1
   - ☐ Most of the time 2
   - ☐ A good bit of the time 3
   - ☐ Some of the time 4
   - ☐ A little of the time 5
   - ☐ Hardly any of the time 6
   - ☐ None of the time 7
9. **How much of the time during the past 2 weeks have you been troubled by a feeling of having to go to the bathroom even though your bowels were empty?**
   - ☐ All of the time 1
   - ☐ Most of the time 2
   - ☐ A good bit of the time 3
   - ☐ Some of the time 4
   - ☐ A little of the time 5
   - ☐ Hardly any of the time 6
   - ☐ None of the time 7
10. **How often during the past 2 weeks have you felt angry as a result of your bowel problem?**
   - ☐ All of the time 1
   - ☐ Most of the time 2
   - ☐ A good bit of the time 3
   - ☐ Some of the time 4
   - ☐ A little of the time 5
   - ☐ Hardly any of the time 6
   - ☐ None of the time 7

Figure 1. Short Inflammatory Bowel Disease (IBD) Questionnaire Questions with answer score values.

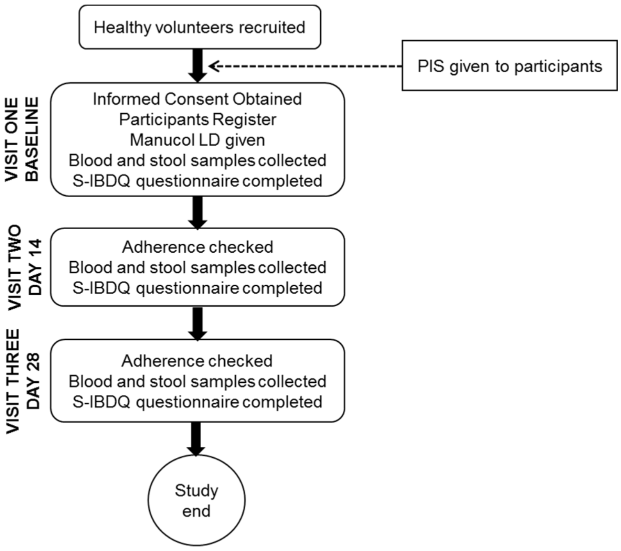

Figure 2. Flow-diagram of healthy volunteer involvement within the study. Patient information sheet (PIS).

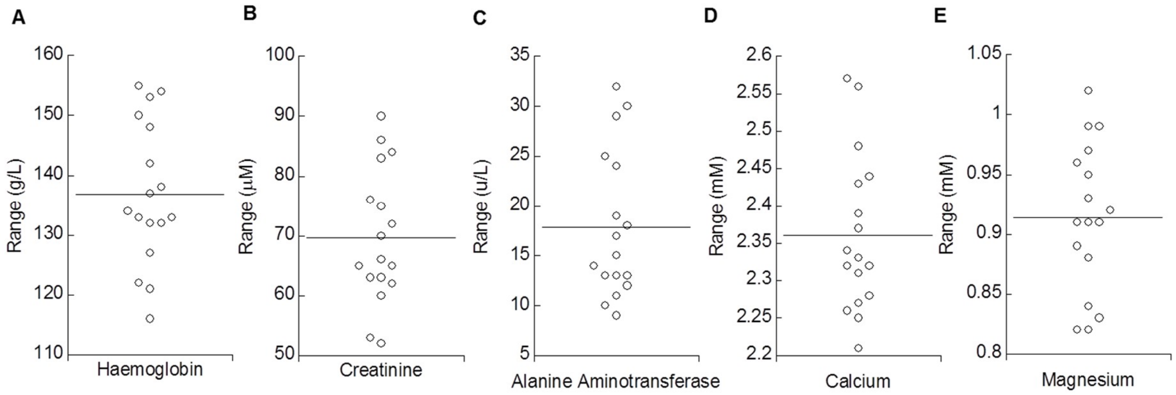

Figure 3. Visit one (baseline) haematological measurements for (A) haemoglobin, (B) creatinine, (C) alanine aminotransferase, (D) calcium and (E) magnesium. Each point represents a healthy participant, and the mid-line denotes the mean.

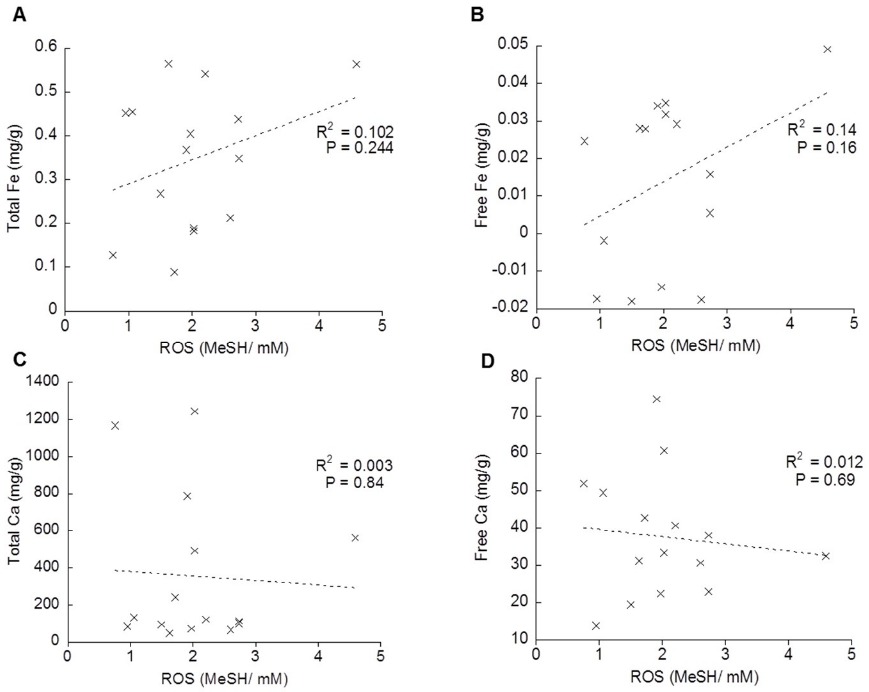

Figure 4. Correlation plots of both free and total stool iron and calcium concentrations vs. stool ROS concentrations. (A) Total Fe vs. ROS, (B) free Fe vs. ROS, (C) total Ca vs. ROS and (D) free Ca vs. ROS. Dashed-lines represent lines of best fit with associated R^2 value and P value representing statistical significance of the observed correlation.

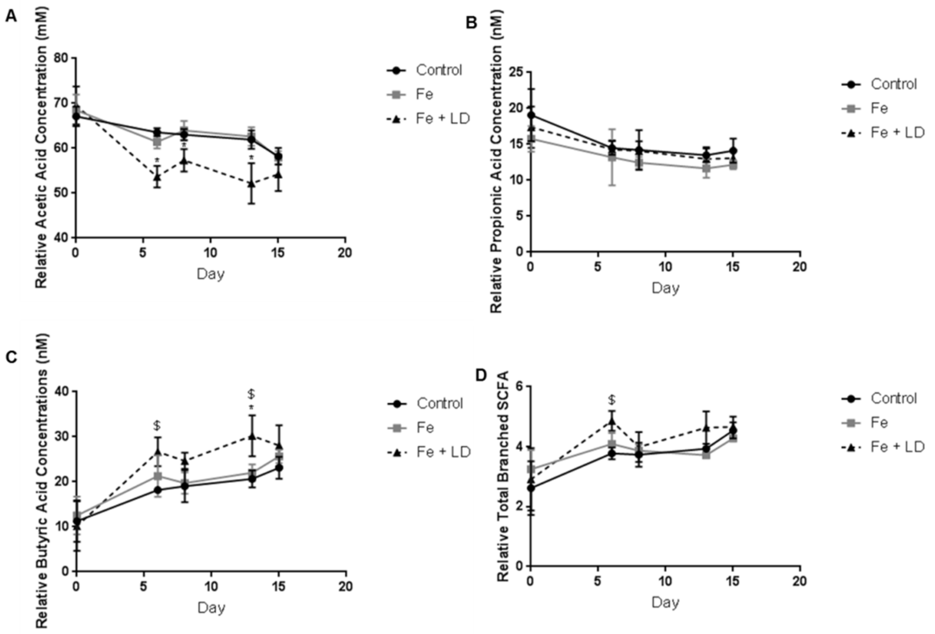

Figure 5. Relative short chain fatty acid (SCFA) changes with respect to total SCFA concentrations. Acetic acid (A), propionic acid (B), butyric acid (C) and total branched SCFAs were measured over the time course of the experiment. Each point represents the mean values with error bars denoting standard deviations in the error. Statistical significance (using 1way ANOVA tests, where $p < 0.05$) is represented with an * for Fe + LD vs. Fe alone and $ for Fe + LD vs. Control with $n = 3$ for each mean value.
